# Adverse events reporting of Etelcalcetide: a real-word analysis from FAERS database

**DOI:** 10.1080/20523211.2025.2479072

**Published:** 2025-03-31

**Authors:** Dongdong Zhang, Ying Cai, Yixin Sun, Peiji Zeng, Wei Wang, Wenhui Wang, Chengfu Cai

**Affiliations:** a College of Otorhinolaryngology Head and Neck Surgy, Xiamen Hospital of Traditional Chinese Medicine, Xiamen, People's Republic of China; b School of Medicine, Xiamen University, Xiamen, People's Republic of China

**Keywords:** Etelcalcetide, FAERS, ROR, BCPNN, EBGM

## Abstract

**Background:**

This study's main goal was to closely monitor and record adverse events (AEs) related to the medication Etelcalcetide, which is used to treat secondary hyperparathyroidism (SHPT, which is defined as elevated parathyroid hormone (PTH) levels in response to abnormalities in the calcium, phosphate, and vitamin D homeostasis). Optimising patient safety and offering evidence-based recommendations for the proper use of this medication are the ultimate goals.

**Methods:**

A thorough collection and analysis of reports from the FDA Adverse Event Reporting System (FAERS) database was conducted, encompassing the first quarter of 2014 to the first quarter of 2024. Robust algorithms including as ROR, PRR, BCPNN, and EBGM were employed for proportional analysis, enabling efficient data mining to measure signals linked to AEs related to Etelcalcetide.

**Results:**

Based on the reports gathered, the number of patients in the Etelcalcetide population was found to be 2,472 (5,435 AEs). As expected, the study's findings revealed the occurrence of Decreased blood calcium, Hypophosphatemia, among other AEs, which are in line with the instructions in the medication insert. Furthermore, unforeseen major AEs were noted at the preferred term (PT) level. These included hunt stenosis, Shunt aneurysm, Shunt occlusion, Shunt infection and Peripheral arterial occlusive disease (PAOD) and so on. These results point to the possibility of AEs that are not presently listed in the medication description.

**Conclusion:**

This work successfully identified previously unidentified and novel signals linked to AEs associated with the administration of Etelcalcetide, offering crucial insights into the intricate relationship between AEs and Etelcalcetide use. In the context of Etelcalcetide therapy, the study's findings highlight the vital significance of diligent surveillance and ongoing monitoring for the prompt detection and efficient management of AEs and to enhance overall patient safety and well-being.

## Background

Etelcalcetide (Etel), a second-generation agonists of the calcium-sensing receptor (CaSR), was approved by the U.S. Food and Drug Administration (FDA) in 2017 for the treatment of secondary hyperparathyroidism (SHPT), a condition characterised by elevated levels of parathyroid hormone (PTH) levels due to disruptions in calcium, phosphate, and vitamin D. (D'Marco et al., 2023; Patel & Bridgeman, 2018). According to the Kidney Disease: Improving Global Outcomes (KDIGO) guidelines, it is primarily recommended for Chronic Kidney Disease (CKD) Stage 5D (end-stage renal disease, ESRD) to reduce PTH levels in dialysis-dependent patients with SHPT (Massy et al., 2014).

Etel acts as a G protein-coupled receptor agonist, inhibits PTH secretion by covalently inding D-cysteine to cysteine 482 of the CaSR, thereby functioning as an orthotropic activator to suppress PTH secretion, reduce serum calcium, and attenuate post-dialysis phosphate elevation (Cozzolino et al., 2015; Walter et al., 2013). Unlike the first-generation calcimimetic cinacalcet, Etel does not require extracellular calcium bound to CaSR activation (Block et al., 2004; Breitwieser, 2014). Its longer half-life allows for a three-times-weekly dosing regimen, reducing overall drug burden (Friedl & Zitt, 2018). Preclinical and clinical studies demonstrate that Etel reduces bone turnover and cortical porosity(Ct.Po) improving trabecular bone structure in CKD-SHPT patients via PTH suppression (Khairallah et al., 2023; Li et al., 2017; Ruderman et al., 2020; Shigematsu et al., 2018).

With the increasing use of Etel, its safety and potential adverse effects have raised concerns among researchers and clinicians. In pooled analyses NCT01785849 and NCT01788046, patients treated with Etel reported more frequent AEs, including hypocalcemia, muscle cramps, and gastrointestinal symptoms in Etle-treated patients compared placebo (Blair, 2016). If intact parathyroid hormone (iPTH) suppression below 100 pg/mL may increase the risk of adynamic bone disease and QTc interval prolongation (>500 ms; corrected via Fridericia’s formula, QTcF) (Blair, 2016), underscoring the need for real-world safety evaluations.

The FDA Adverse Event Reporting System (FAERS) is one of the world's largest databases for monitoring adverse drug events, providing a rich resource of data for studying drug safety (Mikami et al., 2021; Zhao et al., 2023). The FAERS database provided the data for this study, which is updated on a quarterly basis. Reports from patients, pharmaceutical firms, healthcare providers, and other organisations covering adverse event data for a variety of drugs are included in the database. These reports cover medication errors, drug abuse, AEs that may arise with drug use, and other drug-related incidents. By applying signal mining techniques, we comprehensively assessed Etel-associated AEs to identify potential safety risks and inform clinical decision-making.

## Methods

### Study design and data sources

**Figure 1.**
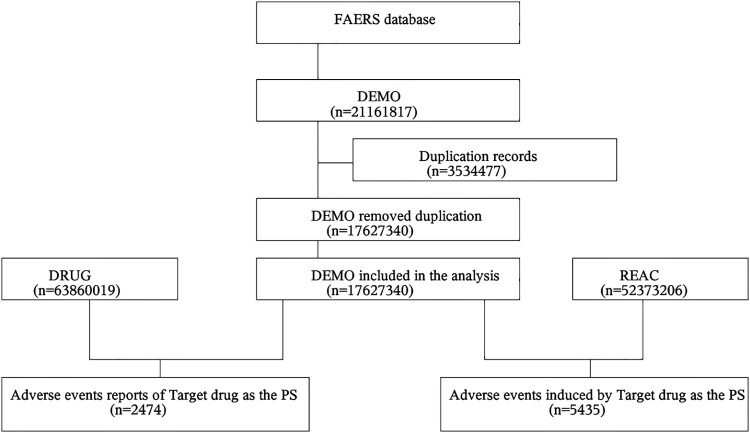
Multistep process of data extraction, processing, and analysis from the food and drug administration adverse event reporting system database.

2,474 patients (5,435 adverse events) were included in the population of patients receiving the target medication Etel in the raw data utilised for data mining, which covered the period from Q1 2014 to Q1 2024. INDEX (PROD_AI, ‘ETELCALCETIDE'), INDEX (DRUGNAME, ‘ETELCALCETIDE’), INDEX (DRUGNAME, ‘AMG 416'), or INDEX (DRUGNAME, ‘PARSABIV') were the screening criteria. Since the FAERS database gathers data through voluntary submissions and contains some duplicate or withdrawn/deleted reports, this study closely adhered to the FDA's official guidance document for data cleansing. In order to clean the data, the first step was to de-duplicate the reports using the FDA-recommended method. Next, the reports were sorted based on CASEID, FDA_DT, and PRIMARYID. The reports were then kept in order of CASEID, FDA_DT, and PRIMARYID. The report with the highest value of FDA_DT was kept for reports that had the same CASEID, and the report with the highest value of FDA_DT was kept for reports that had the same CASEID and FDA_DT. The largest FDA_DT value was kept for reports that shared the same CASEID, and the largest PRIMARYID value was kept for reports that shared the same CASEID and FDA_DT. English version 26.0, and the MedDRA's principal system organ classifications (SOCs) were assigned to these Preferred Terms (PTs). If two or more PTs were included in a single report, they were regarded as distinct AEs. Figure 1 displays the data screening flow chart.

### Signal detection and analysis

To identify possible Etel–AEs correlations, pharmacovigilance currently does a disproportionality analysis. Four methods were used in response to the disproportionality analysis: reporting odds ratio (ROR), the proportional reporting ratio (PRR), the Bayesian confidence propagation neural network (BCPNN) (Lindquist et al., 2000; Sun et al., 2019), and the multi-item gamma Poisson shrinker (MGPS). These methods enable systematic comparison AE reporting rates between Etel and all other drugs in the FAERS database (Hauben et al., 2021). As detailed in Supplemental Table 1, the algorithms quantify signal strengths through standardised formulas and thresholds, providing robust evidence for evaluating Etel's safety profile

## Results

### Basic information

**Figure 2.**
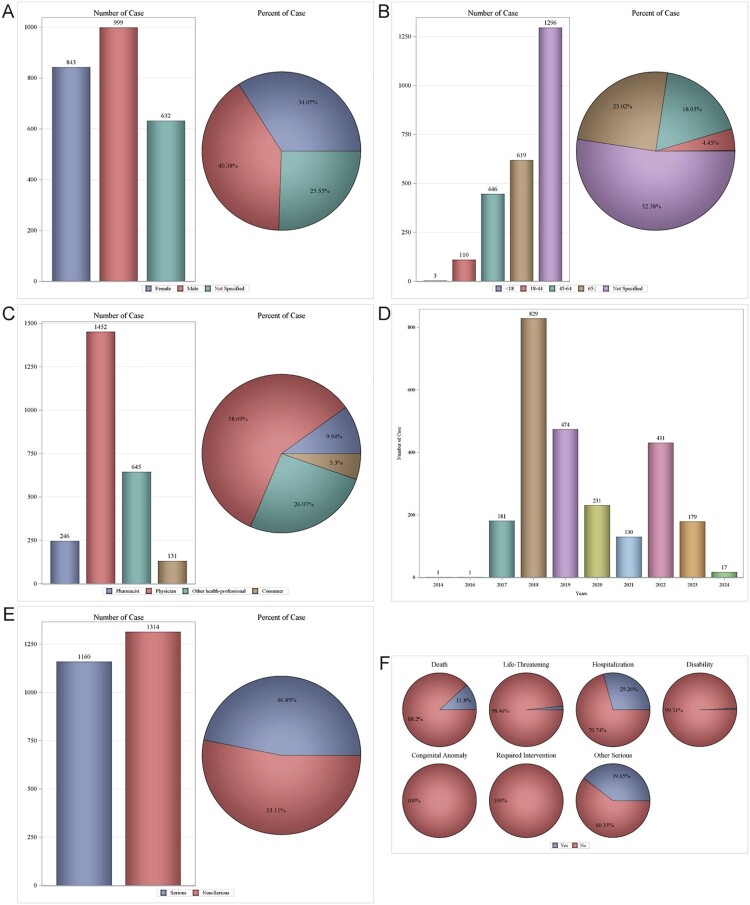
Clinical characteristics of Etelcalcetide associated reports from the FAERS database. (A) gender; (B) age; (C) reporters; (D) years; (E) serious reports; (F) outcomes.

In the FAERS database, 2472 patients experienced 5435 AEs attributed to Etel, with a mean of 2.2 AEs per patient. Gender distribution showed a slight male predominance (40.38%, n = 999) compared to females (34.07%, n = 843) (Figure 2A). Age stratification revealed the highest proportion in patients ≥65 years (25.02%, n = 619), followed by 45–64 years (18.03%, n = 446), with a mean age = 63.61 ± 14.15 years (Figure 2B). Reporters were predominantly Physicians with more than 50%, followed by Other health-professionals at 26.07% (Figure 2C). The highest number of cases were reported in 2018, with more than 30% (Figure 2D). Geographically, the United States of America was the most reported country at 1580 cases (63.86%) and Japan at 768 cases (31.04%) (Supplemental Table 2). AE severity classification indicated non-serious AEs(53.11%) and serious AEs (46.89%) (Figure 2E). Outcomes were mainly Hospitalization (Initial or prolonged) (n = 724, 29.26%), death (n = 292, 11.80%) and other (n = 981, 39.65%) (Figure 2F).

### Etelcalcetide signal mining

After screening the signals of AEs mainly suspected to be Etel, we filtered them using the four most stringent methods (ROR, PRR, BCPNN, and MGPS) to produce results involving 26 SOCs (Table 1). Among these reports, the most frequently reported AEs were all types of Injury, poisoning and procedural complications (n = 1207, ROR 2.53 (2.37–2.69), PRR 2.19 (2.08–2.3), χ2 865.64, IC 1.13 (1.04–1.22), EBGM 2.19 (2.05–2.33)), Gastrointestinal disorders (n = 671, ROR 1.51 (1.4–1.64), PRR 1.45 (1.35–1.56), χ2 102.17, IC 0.54 (0.42–0.65), EBGM 1.45 (1.34–1.57)) and General disorders and administration site conditions (n = 529, ROR 0.51 (0.47–0.56), PRR 0.56 (0.51–0.61), χ2 224.16, IC −0.84 (−0.97–0.71), EBGM 0.56 (0.51–0.61)). It is worth noting that in addition to the AEs explicitly mentioned in the drug labelling, several frequent AEs were identified in this study, such as various types of Injuries, poisoning and procedural complications, Investigations, Infections and infestations, Skin and subcutaneous tissue disorders, Vascular disorders, Respiratory, thoracic and mediastinal disorders, Neoplasms benign, malignant and unspecified (incl cysts and polyps), which require special attention in the clinical setting.

**Table 1.** The affected systems/organs of Etelcalcetide.

System organ class	SOC code	Case reports	ROR(95%CI)	PRR(95%CI)	χ2	IC(95%CI)	IC signal strength	EBGM(95%CI)
Injury, poisoning and procedural complications	10022117	1207	2.53(2.37–2.69)	2.19(2.08–2.3)	865.64	1.13(1.04–1.22)	+	2.19(2.05–2.33)
Gastrointestinal disorders	10017947	671	1.51(1.4–1.64)	1.45(1.35–1.56)	102.17	0.54(0.42–0.65)	+	1.45(1.34–1.57)
General disorders and administration site conditions	10018065	529	0.51(0.47–0.56)	0.56(0.51–0.61)	224.16	−0.84(−0.97–0.71)	−	0.56(0.51–0.61)
Investigations	10022891	516	1.59(1.46–1.75)	1.54(1.42–1.67)	103.61	0.62(0.49–0.75)	+	1.54(1.4–1.68)
Infections and infestations	10021881	404	1.46(1.32–1.61)	1.42(1.3–1.56)	53.92	0.51(0.36–0.66)	+	1.42(1.29–1.58)
Nervous system disorders	10029205	368	0.78(0.7–0.86)	0.79(0.72–0.87)	22.11	−0.34(−0.49–0.18)	−	0.79(0.71–0.88)
Cardiac disorders	10007541	335	2.4(2.15–2.68)	2.31(2.08–2.56)	255.75	1.21(1.04–1.36)	+	2.31(2.07–2.58)
Musculoskeletal and connective tissue disorders	10028395	235	0.83(0.73–0.94)	0.83(0.74–0.94)	8.21	−0.26(−0.45–0.07)	−	0.83(0.73–0.95)
Metabolism and nutrition disorders	10027433	221	1.9(1.66–2.18)	1.87(1.64–2.13)	91.12	0.90(0.70–1.09)	+	1.87(1.63–2.14)
Skin and subcutaneous tissue disorders	10040785	199	0.67(0.58–0.77)	0.68(0.59–0.78)	31.32	−0.55(−0.76–0.34)	−	0.68(0.59–0.79)
Vascular disorders	10047065	138	1.19(1–1.4)	1.18(1–1.39)	3.90	0.24(−0.01–0.49)	−	1.18(1–1.4)
Respiratory, thoracic and mediastinal disorders	10038738	107	0.41(0.34–0.49)	0.42(0.35–0.5)	90.90	−1.26(−1.53–0.97)	−	0.42(0.35–0.51)
Neoplasms benign, malignant and unspecified (incl cysts and polyps)	10029104	79	0.54(0.43–0.67)	0.54(0.44–0.68)	31.05	−0.88(−1.2–0.55)	−	0.54(0.44–0.68)
Psychiatric disorders	10037175	74	0.23(0.18–0.29)	0.24(0.19–0.3)	190.86	−2.07(−2.39–1.72)	−	0.24(0.19–0.3)
Hepatobiliary disorders	10019805	61	1.23(0.95–1.58)	1.23(0.96–1.57)	2.56	0.29(−0.08–0.66)	−	1.23(0.95–1.58)
Surgical and medical procedures	10042613	56	0.77(0.6–1.01)	0.78(0.6–1.01)	3.63	−0.36(−0.74–0.03)	−	0.78(0.6–1.01)
Eye disorders	10015919	52	0.48(0.36–0.63)	0.48(0.37–0.63)	29.30	−1.05(−1.43–0.64)	−	0.48(0.37–0.64)
Blood and lymphatic system disorders	10005329	44	0.48(0.35–0.64)	0.48(0.36–0.65)	24.94	−1.05(−1.47–0.61)	−	0.48(0.36–0.65)
Product issues	10077536	27	0.31(0.21–0.45)	0.31(0.22–0.46)	41.22	−1.67(−2.18–1.09)	−	0.31(0.21–0.46)
Immune system disorders	10021428	26	0.43(0.3–0.64)	0.44(0.3–0.64)	19.03	−1.19(−1.72–0.61)	−	0.44(0.3–0.64)
Renal and urinary disorders	10038359	25	0.24(0.16–0.35)	0.24(0.16–0.35)	61.75	−2.06(−2.59–1.45)	−	0.24(0.16–0.35)
Endocrine disorders	10014698	19	1.39(0.88–2.18)	1.39(0.88–2.17)	2.04	0.47(−0.2–1.09)	−	1.39(0.88–2.17)
Ear and labyrinth disorders	10013993	15	0.63(0.38–1.05)	0.63(0.38–1.05)	3.20	−0.66(−1.35–0.1)	−	0.63(0.38–1.05)
Reproductive system and breast disorders	10038604	14	0.28(0.17–0.48)	0.28(0.17–0.48)	25.37	−1.81(−2.49–1)	−	0.28(0.17–0.48)
Social circumstances	10041244	11	0.44(0.24–0.79)	0.44(0.24–0.79)	8.00	−1.19(−1.96–0.29)	−	0.44(0.24–0.79)
Congenital, familial and genetic disorders	10010331	2	0.12(0.03–0.48)	0.12(0.03–0.48)	12.91	−3.06(−4.22–0.89)	−	0.12(0.03–0.48)

After comprehensive evaluation, a total of 117 significant PTs were identified that met the criteria of all 4 algorithms. These PTs were ranked according to the most stringent algorithm, EBGM, and the top 30 PTs are shown in Table 2. Shunt stenosis in signal intensity (n = 200, ROR 30313.28 (22920.61–40090.34), PRR 29197.83 (22134.42–38515.28), χ2 1448823.07, IC 12.82 (7.34–7.88), EBGM 7245.33 (5478.37–9582.19)), Shunt aneurysm (n = 10, ROR 6895.03 (3061.32–15529.70), PRR 6882.35 (3058.36–15487.62), χ2 40135.37, IC 11.97 (2.41–4.50), EBGM 4015.12 (1782.67–9043.26)), Shunt occlusion (n = 126, ROR 3970.77 (3223.37–4891.47), PRR 3878.74 (3159.57–4761.61), χ2 348273.79, IC 11.43 (6.63–7.22), EBGM 2765.77 (2245.18–3407.06)) and Shunt infection (n = 14, ROR 735.01 (426.6–1266.40), PRR 733.12 (426.05- 1261.50), χ2 9511.99, IC 9.41 (3.10–4.65), EBGM 681.35 (395.45–1173.95)) were the most significant, and the signals of these new, clinically valuable potential AEs were not consistent with drug labelling content. Also consistent with clinical observations and drug labelling content and occurring with relatively high frequency were Blood parathyroid hormone increased (n = 121, ROR 481.96 (400.77–579.58), PRR 471.25 (393.41–564.49), χ2 54134.52, IC 8.81 (6.32–6.86), EBGM 449.32 (373.64–540.34)), Blood calcium decreased (n  = 105, ROR 106.75 (87.91–129.63), PRR 104.71 (86.55- 126.68), χ2 10671.56, IC 6.69 (5.43–6.00), EBGM 103.59 (85.31–125.8)), Hypocalcaemia (n  = 120, ROR 75.39 (62.87–90.40), PRR 73.75 (61.75–88.08), χ2 8548.65, χ2 8548.65, EBGM 103.59 (85.31–88.8)), Blood parathyroid hormone abnormal (n  = 30, ROR 512.63 (354.74–740.78), PRR 509.80 (353.47–735.28), χ2 14468.79, IC 8.92 (4.33–5.40), EBGM 484.24 (335.1–699.75)), Blood parathyroid hormone decreased (n  = 24, ROR 189.61(126.48–284.24), PRR 188.77(126.15–282.49), χ2 4396.65, IC 7.53(3.88–5.05), EBGM 185.16(123.52–277.58), Blood phosphorus increased (n = 22, ROR 97.40 (63.95–148.37), PRR 97.01 (63.8–147.52), χ2 2069.79, IC 6.59 (3.62–4.83), EBGM 96.06 (63.06–146.31)) and Blood calcium abnormal (n = 20, ROR 138.95-5.05, EBGM 185.16 (123.52–277.58)), which are also based on the mechanism of action of Etel AEs that may occur. Notably, Peripheral arterial occlusive disease(PAOD) (n = 60, ROR 160.01 (123.81–206.80), PRR 158.26 (122.79–203.96), χ2 9225.01, IC 7.28 (5.09- 5.83), EBGM 155.72 (120.49–201.25)) occurred with relatively high frequency, and thus special care needs to be taken with these potential AEs when using Etel in clinical practice.

**Table 2.** The top 30 signal strength of AEs of Etelcalcetide ranked by EBGM at the PTs level.

SOC	PTs	Case reports	ROR(95%CI)	PRR(95%CI)	χ2	IC(95%CI)	IC signal strength	EBGM(95%CI)
Injury, poisoning and procedural complications	Shunt stenosis	200	30313.28(22920.61–40090.34)	29197.83(22134.42–38515.28)	1448823.07	12.82(7.34–7.88)	+++	7245.33(5478.37–9582.19)
Injury, poisoning and procedural complications	Shunt aneurysm	10	6895.03(3061.32–15529.70)	6882.35(3058.36–15487.62)	40135.37	11.97(2.41–4.50)	++	4015.12(1782.67–9043.26)
Injury, poisoning and procedural complications	Shunt occlusion	126	3970.77(3223.37–4891.47)	3878.74(3159.57–4761.61)	348273.79	11.43(6.63–7.22)	+++	2765.77(2245.18–3407.06)
Infections and infestations	Shunt infection	14	735.01(426.6–1266.40)	733.12(426.05–1261.50)	9511.99	9.41(3.10–4.65)	+++	681.35(395.45–1173.95)
Vascular disorders	Dialysis hypotension	4	676.66(245.43–1865.56)	676.16(245.42–1862.89)	2519.82	9.30(0.97–3.65)	+	631.89(229.19–1742.13)
Infections and infestations	Puncture site infection	3	657.31(204.04–2117.54)	656.95(204.05–2115.10)	1839.44	9.26(0.49–3.49)	+	615.08(190.93–1981.49)
Investigations	Blood parathyroid hormone abnormal	30	512.63(354.74–740.78)	509.80(353.47–735.28)	14468.79	8.92(4.33–5.40)	+++	484.24(335.1–699.75)
Investigations	Blood parathyroid hormone increased	121	481.96(400.77–579.58)	471.25(393.41–564.49)	54134.52	8.81(6.32–6.86)	+++	449.32(373.64–540.34)
Infections and infestations	Hepatic cyst infection	3	352.70(111.41–1116.62)	352.51(111.42–1115.32)	1014.43	8.41(0.51–3.46)	+	340.10(107.43–1076.73)
Investigations	Adjusted calcium decreased	3	321.35(101.69–1015.52)	321.18(101.7–1014.34)	926.65	8.28(0.51–3.46)	+	310.85(98.36–982.33)
Injury, poisoning and procedural complications	Shunt malfunction	7	284.95(134.31–604.54)	284.59(134.27–603.19)	1921.39	8.11(1.93–4.00)	++	276.45(130.31–586.5)
Injury, poisoning and procedural complications	Arteriovenous fistula site haemorrhage	4	245.67(91.03–662.97)	245.48(91.03–662.01)	949.76	7.90(0.99–3.61)	+	239.41(88.71–646.09)
Infections and infestations	Renal cyst infection	3	222.47(70.8–699.04)	222.35(70.81–698.23)	646.16	7.76(0.52–3.44)	+	217.36(69.18–682.97)
Musculoskeletal and connective tissue disorders	Hungry bone syndrome	3	222.47(70.8–699.04)	222.35(70.81–698.23)	646.16	7.76(0.52–3.44)	+	217.36(69.18–682.97)
Surgical and medical procedures	Parathyroidectomy	9	219.35(113.23–424.92)	218.98(113.16–423.76)	1909.51	7.74(2.34–4.19)	++	214.14(110.54–414.83)
Investigations	Blood phosphorus abnormal	8	200.51(99.51–404.03)	200.21(99.46–403.03)	1553.48	7.62(2.14–4.09)	++	196.16(97.35–395.26)
Investigations	Blood parathyroid hormone decreased	24	189.61(126.48–284.24)	188.77(126.15–282.49)	4396.65	7.53(3.88–5.05)	+++	185.16(123.52–277.58)
Vascular disorders	Peripheral arterial occlusive disease	60	160.01(123.81–206.80)	158.26(122.79–203.96)	9225.01	7.28(5.09–5.83)	+++	155.72(120.49–201.25)
Infections and infestations	Diabetic gangrene	4	150.66(56.1–404.65)	150.55(56.09–404.06)	585.09	7.21(0.98–3.59)	+	148.25(55.2–398.17)
Investigations	Blood calcium abnormal	20	138.95(89.29–216.22)	138.44(89.11–215.08)	2690.32	7.09(3.56–4.83)	+++	136.49(87.71–212.4)
Nervous system disorders	Putamen haemorrhage	3	114.31(36.61–356.94)	114.25(36.61–356.52)	332.84	6.82(0.51–3.42)	+	112.93(36.17–352.6)
Investigations	Blood calcium decreased	105	106.75(87.91–129.63)	104.71(86.55–126.68)	10671.56	6.69(5.43–6.00)	+++	103.59(85.31–125.8)
Cardiac disorders	Aortic valve stenosis	16	102.67(62.69–168.14)	102.37(62.6–167.41)	1589.19	6.66(3.17–4.58)	+++	101.30(61.85–165.9)
Investigations	Blood phosphorus increased	22	97.40(63.95–148.37)	97.01(63.8–147.52)	2069.79	6.59(3.62–4.83)	+++	96.06(63.06–146.31)
Metabolism and nutrition disorders	Hypocalcaemia	120	75.39(62.87–90.40)	73.75(61.75–88.08)	8548.65	6.19(5.25–5.78)	+++	73.20(61.04–87.77)
Injury, poisoning and procedural complications	Heat illness	4	69.87(26.12–186.90)	69.82(26.12–186.63)	269.39	6.12(0.94–3.54)	+	69.33(25.92–185.44)
Infections and infestations	Pyoderma	3	66.79(21.45–207.98)	66.76(21.45–207.74)	192.98	6.05(0.49–3.39)	+	66.30(21.29–206.46)
Metabolism and nutrition disorders	Calciphylaxis	11	64.01(35.36–115.89)	63.89(35.33–115.52)	676.46	5.99(2.52–4.19)	++	63.47(35.06–114.91)
Nervous system disorders	Thalamus haemorrhage	5	61.27(25.42–147.68)	61.22(25.42–147.42)	294.29	5.93(1.29–3.65)	+	60.84(25.24–146.63)
Neoplasms benign, malignant and unspecified (incl cysts and polyps)	Parathyroid tumour benign	4	59.89(22.4–160.12)	59.85(22.4–159.89)	230.03	5.89(0.93–3.52)	+	59.48(22.25–159.03)

**Figure 3.**
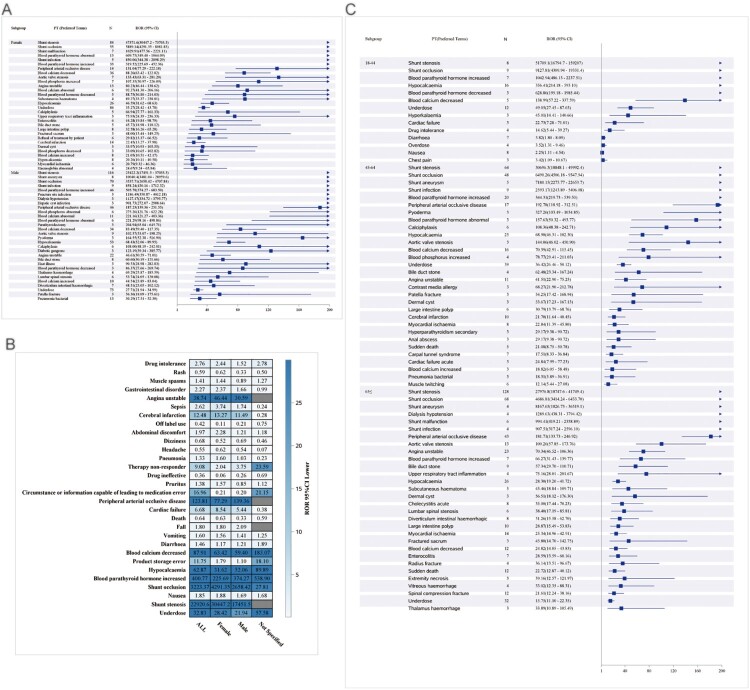
The PT results for Etelcalcetide subgroup analysis in ROR. (A–B) gender; (C) age.

Immediately following this, we further refined our results by subgroup analysis, with results from the gender subgroup suggesting that Shunt stenosis and Shunt occlusion occur predominantly in males, but signal intensity is weaker in females. Blood parathyroid hormone increased, Peripheral arterial occlusive disease, Hypocalcemia, Shunt aneurysm, and Shunt infection occured predominantly in males and has a stronger signal intensity than females. Blood calcium decreased and Blood parathyroid hormone abnormal occurred predominantly in females and had a stronger signal intensity than in males (Figure 3A–B). Age grouping revealed that Blood parathyroid hormone increased and Blood calcium decreased were mainly in the 45–64 age group and had a stronger signal intensity, while Shunt aneurysm and Shunt infection were relatively weaker. Shunt stenosis, Shunt occlusion, Hypocalcemia, and Peripheral arterial occlusive disease occurred mainly in people older than or equal to 65 years of age, but the signals were relatively weak in the 45–64 age group (Figure 3C).

### Onset time of Etelcalcetide-related AEs

**Figure 4.**
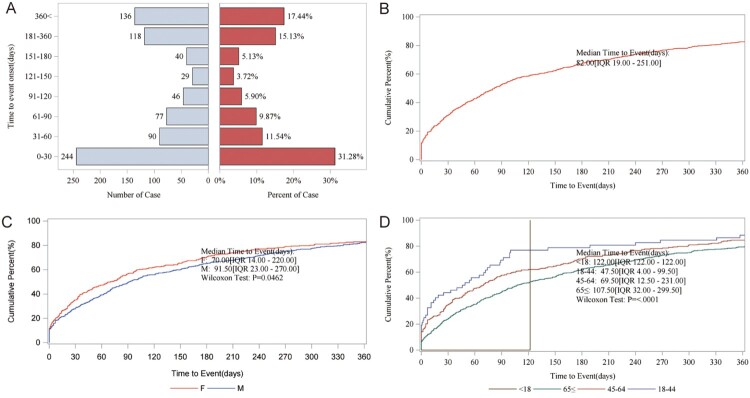
AE occurrence time-medication date subgroup analysis. (A) AE occurrence time-medication date; (B) median time to event; (C) gender; (D) age.

Time of AE occurrence-medication date not included Unspecified was mostly clustered in the range of 0–30 days (31.28%), then >360 days (n = 136, 17.44%), and 181–360 days (n = 118, 15.13%) (Figure 4A). Subsequent subgroup analysis revealed that the median AE occurrence time-medication date was 82d (Figure 4B). The gender subgroup showed a significant difference (*p *= 0.0462), and the age subgroup showed an even more substantial difference (*p*<0.0001) (Figure 4C–D).

## Discussion

Secondary hyperparathyroidism (SHPT), a severe complication of progressive chronic kidney disease (CKD), affecting approximately 2 million dialysis patients worldwide (Liyanage et al., 2015; Tomasello, 2008). Epidemiological studies indicate that the prevalence of SHPT ranges from 30% to 49% in European dialysis populations, while approximately 54% of adult dialysis patients in the United States are affected (Hedgeman et al., 2015). The incidence of SHPT escalates with declining renal function: it affects about 20% of patients at CKD stage G3a and exceeds 80% by CKD stage G5 (end-stage renal disease) (Cunningham et al., 2011). Observational studies further demonstrate that severely elevated PTH levels are associated with increased risks of all-cause mortality and cardiovascular events in dialysis patients. Parathyroidectomy, by markedly reducing serum PTH levels, has been shown to improve clinical outcomes in this population (Floege et al., 2011; Kalantar-Zadeh et al., 2006; Tentori et al., 2015).

Etelcalcetide is the first intravenous calcium mimetic approved for the treatment of SHPT in hemodialysis patients in Europe, the United States and Japan (Hamano et al., 2017). Etel package inserts and a number of clinical studies have concluded that Etel's AEs are significantly different from those in the placebo group, highlighting the need to elucidate its safety in real-world settings (Block et al., 2017; Bushinsky et al., 2016). Our study is the first extensive systematic and in-depth pharmacovigilance analysis of Etel using the FAERS database, which was compared with previous clinical studies to identify potential AE risks.

The clinical management of SHPT requires integrated decision-making based on evidence-based guideline, such as the 2017 KDIGO CKD–mineral and bone disorder (MBD) recommendations, though their applicability is limited by low evidence grades (Kidney Dis Improving, 2017; Kidney Disease: Improving Global Outcomes, 2009). Current consensus positions calcimimetics (e.g. cinacalcet, etelcalcetide) as first-line agents for reducing PTH levels in dialysis patients (Bushinsky et al., 2015; Conigrave, 2016; Goodman et al., 2002; Walter et al., 2013). However, real-world implementation faces barriers such as reimbursement policies (e.g. the U.S. ESRD bundled payment system) (Stephens et al., 2011)), which may delay calcimimetic initiation. Early etelcalcetide intervention (PTH <600 pg/mL) is critcial for optimal outcomes (Cunningham et al., 2019).

With Etel being available in many countries around the world, reports of FAERS AEs with Etel as the first suspected drug are increasing every year. Our study counted baseline information on patients, and there was no significant difference in Etel use between Male and Female, and the age level was highest at ≥65 years of age, which are also more consistent with pharmacoepidemiologic findings. The reporters were predominantly Physicians, accounting for more than 50% of the total. This is mainly due to the fact that the FAERS database constitutes a self-reporting system, and the most important part of the reporting is done by Physicians (Li et al., 2021). Outcome is primarily Hospitalization – Initial or Prolonged may be due to the occurrence of more serious adverse events that have to be Hospitalized to be resolved. 31.28% of AEs occurred within 30 days of administration, which is a key observation indicating the need for vigilant monitoring during this period. Subgroup analysis revealed significant differences among age groups, which may be attributed to the fact that Etel use also varies among different populations, resulting in this phenomenon.

In our study, we found a large number of PT signals associated with primary diseases, such as Blood parathyroid hormone increase, Blood calcium decreased, Hypocalcaemia, Blood parathyroid hormone abnormal, Blood parathyroid hormone decreased, Blood phosphorus increased, Blood calcium abnormal, these are also possible AEs based on the mechanism of action of Etel. Abnormalities in calcium, phosphorus, and PTH, a hallmark of CKD-MBD markers are associated with poor outcomes in patients on maintenance dialysis (Block et al., 1998; Kidney Dis Improving, 2017; Tentori et al., 2015). In patients with advanced CKD, the development of SHPT leads to CKD-MBD and disorders of circulating markers of mineral metabolism, as well as skeletal and cardiovascular physiology (Cunningham et al., 2011). Etel can metabolically modulate CaR, enhance extracellular calcium receptor activation, and decrease PTH secretion, leading to decreased calcium and attenuated post-dialysis phosphate elevation (Ye et al., 2018). Generally speaking, AEs data mining can identify unusual or delayed AEs and support pre-marketing clinical investigations of pharmaceuticals. However, because of the influence of the underlying patient disease, the quantity of target medications, and the quantity of target AEs, good signals could be skewed or overlooked. This is the most typical way that AEs data mining research are limited (Bihan et al., 2020). Examples include Nausea, Vomiting, Diarrhea in Gastrointestinal disorders (Block et al., 2017), Prolonged QT interval in Cardiac disorders in labelling and safety data from clinical trials (Kahn et al., 1987), but our data study did not find these associated positive AEs, or were not major AEs.

In our analysis, although most of the AEs were more consistent with labelling and safety data from clinical trials, we still identified other important AE signals that had not previously been explicitly reported in regulatory trials, such as the more significant signal intensities of Shunt stenosis, Shunt aneurysm, Shunt occlusion, and Shunt infection. Shunt is a medical device that is used to create a new channel in the body to bypass a blocked or damaged blood vessel or organ. For example, in hemodialysis, an arteriovenous fistula (AVF) or arteriovenous fistula (AVG) may become narrowed due to scar tissue formation or other reasons, interfering with blood flow (Huber et al., 2021; Locatelli & Zoccali, 2015). However, we did not find a relationship between Etel and shunt-related disease. We conjecture that because Etel is used in CKD patients who may have been or will be on hemodialysis, they may have had or may have AVF. Shunt-related disease is one of the complications that hemodialysis patients may encounter, but Etel's use is primarily directed at regulating PTH and blood calcium levels rather than directly contributing to shunt-related disease. PAOD is also an AE that occurs with relatively high frequency. PAOD, also known as peripheral arterial disease (PAD), is a condition in which peripheral arterial vessels are narrowed or occluded due to atherosclerosis, commonly in the lower extremities, and may affect blood flow to the extremities, causing pain, dysfunction, and tissue damage (Cooke & Ma, 1995; Foley et al., 2016; Ingwersen et al., 2023). The relationship between Etel and PAOD is not directly mentioned in the drug's description and labelling. We believe this may be due to the fact that Etel is primarily a SHPT for CKD patients, who may also be at higher risk for PAOD development due to multiple comorbidities and risk factors (e.g. diabetes mellitus, hypertension, hyperlipidemia, etc.), which in turn leads to the AE of PAOD (Asahina et al., 2017; Liao et al., 2015).

This study has certain limitations even though it offers solid scientific support for evaluating Etel's safety from all angles. First, there is a chance of bias and inadequate information because the FAERS database collects data through spontaneous reports. Examples include indication and reporting bias, and it can be difficult to distinguish between AEs brought on by the medication and a worsening of the underlying condition. Furthermore, countries or regions with a large number of reports could be biased in their sample. Furthermore, comorbidities connected to the incidents that were recorded were not taken into account in the data, which could complicate any conclusions drawn about a causal link between Etel and AEs. Thus, it might be difficult to demonstrate a causal relationship with Etel even in the presence of the observed signal. Future research endeavours could investigate the application of a more rigorous prospective technique that blends clinical trials with epidemiologic studies to attain a more thorough and accurate assessment, consequently enhancing the accuracy of safety risk assessments linked to Etel.

## Conclusion

For SHPT, Etelcalcetide, an IV calcimimetic, is the main treatment drug. Even though it has a well-established track record of reducing PTH levels, thorough pharmacovigilance is required to detect and manage any possible AEs related to its administration. This study concluded that PAOD and shunt-related diseases should receive extra attention in addition to common AEs. This publication also includes the median time to onset of labelled and off-label AEs and the outcomes of more thorough subgroup analysis. These details are helpful for physicians and pharmacists to manage Etel safety concerns and optimise pharmaceutical use. Given the exploratory nature of our work, it is important to validate our findings in prospective studies and to elucidate the potential mechanisms and risk factors for Etel in order to explore the impact of Etel on drug utilisation.

## Supplementary Material

Supplement Table 1.doc

Supplement Table 2.doc

## Data Availability

The original contributions presented in the study are included in the article/Supplemental Material; further inquiries can be directed to the corresponding author.
