# Supplementary material for: Adverse events reporting of Etelcalcetide: a real-word analysis from FAERS database
Source: J Pharm Policy Pract. 2025 Mar 31;18(1):2479072. doi: 10.1080/20523211.2025.2479072 (PMC11960307; doi:10.1080/20523211.2025.2479072)
Supplement: Supplement Table 1.doc [file JPPP_A_2479072_SM6706.doc]

|  | PRALSETINIB | Non-PRALSETINIB |  |
| --- | --- | --- | --- |
| Target AEs | a | b | a+b |
| No-target AEs | c | d | c+d |
|  | a+c | b+d | N=a+b+c+d |

**Supplement Table1**. Calculation table

[I] ROR algorithm

| Method | Calculation formula | Threshold value |
| --- | --- | --- |
| ROR | 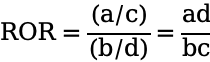  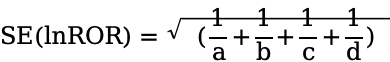  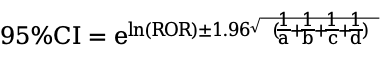 | a≥3 and 95% CI (lower limit) ＞ 1 |

[II] MHRA algorithm

| Method | Calculation formula | Threshold value |
| --- | --- | --- |
| MHRA | 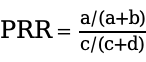  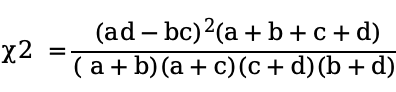 | a≥3 and PRR≥2 and X2≥4 |

[III] BCPNN algorithm

| Method | Calculation formula | Threshold value |
| --- | --- | --- |
| BCPNN | 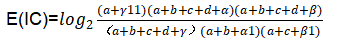  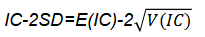 | (-)：IC-2SD ≤0；  (+)：0＜IC-2SD ≤1.5；  (++)：1.5＜IC-2SD ≤3；  (+++)：IC-2SD ＞3 |

[IV] MGPS algorithm

| Method | Calculation formula | Threshold value |
| --- | --- | --- |
| MGPS | 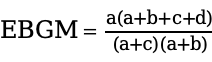  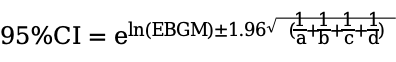 | EBGM05>2 |

Note: ROR, Reporting odds ratio;

CI, Confidence;

a, Number of reports;

PRR, Proportional reporting ratio;

χ2, Chi-squared;

BCPNN, Bayesian confidence propagation neural network;

IC, Information component;

IC025, The lower limit of the 95% two-sided CI of the IC.

EBGM, Empiric Bayes geometric mean;

EBGM05 and the lower 90% one-sided CI of EBGM.
