# Supplementary material for: Adverse events reporting of Etelcalcetide: a real-word analysis from FAERS database
Source: J Pharm Policy Pract. 2025 Mar 31;18(1):2479072. doi: 10.1080/20523211.2025.2479072 (PMC11960307; doi:10.1080/20523211.2025.2479072)
Supplement: Supplement Table 2.doc [file JPPP_A_2479072_SM6705.doc]

**Supplement Table2**. Characteristics of cases of Etelcalcetide usage.

| Characteristics | Number of events (%) |
| --- | --- |
| Gender |  |
| Female(%) | 843(34.07) |
| Male(%) | 999(40.38) |
| Not Specified(%) | 632(25.55) |
| Age |  |
| <18(%) | 3( 0.12) |
| 18-44(%) | 110( 4.45) |
| 45-64(%) | 446(18.03) |
| 65≤(%) | 619(25.02) |
| NotSpecified(%) | 1296(52.38) |
| Report year |  |
| 2014(%) | 1( 0.04) |
| 2016(%) | 1( 0.04) |
| 2017(%) | 181( 7.32) |
| 2018(%) | 829(33.51) |
| 2019(%) | 474(19.16) |
| 2020(%) | 231( 9.34) |
| 2021(%) | 130( 5.25) |
| 2022(%) | 431(17.42) |
| 2023(%) | 179( 7.24) |
| 2024(%) | 17( 0.69) |
| Reporter |  |
| Consumer(%) | 131( 5.30) |
| Other health-professional(%) | 645(26.07) |
| Pharmacist(%) | 246( 9.94) |
| Physician(%) | 1452(58.69) |
| Reported countries（ TOP ten) |  |
| United States of America(%) | 1580(63.86) |
| Japan(%) | 768(31.04) |
| Germany(%) | 38( 1.54) |
| France(%) | 17( 0.69) |
| Spain(%) | 10( 0.40) |
| Austria(%) | 8( 0.32) |
| Belgium(%) | 7( 0.28) |
| Italy(%) | 6( 0.24) |
| Greece(%) | 5( 0.20) |
| China(%) | 5( 0.20) |
| Indications（ TOP ten) |  |
| Hyperparathyroidism secondary(%) | 1294(52.30) |
| Product used for unknown indication(%) | 1074(43.41) |
| Hyperparathyroidism(%) | 29( 1.17) |
| Chronic kidney diseas(%) | 20( 0.81) |
| Blood parathyroid hormone increased(%) | 14( 0.57) |
| Blood parathyroid hormone abnormal(%) | 8( 0.32) |
| Secondary hyperthyroidism(%) | 7( 0.28) |
| End stage renal disease(%) | 6( 0.24) |
| Hyperparathyroidism tertiary(%) | 5( 0.20) |
| Hypoparathyroidism secondary(%) | 5( 0.20) |
| Serious report |  |
| Non-Serious(%) | 1314(53.11) |
| Serious(%) | 1160(46.89) |
| Outcome |  |
| Life-Threatening(%) | 38( 1.54) |
| Hospitalization - Initial or Prolonged(%) | 724(29.26) |
| Disability(%) | 17( 0.69) |
| Death(%) | 292(11.80) |
| Congenital Anomaly(%) | 0( 0.00) |
| Required Intervention to Prevent Permanent Impairment/Damage(%) | 0( 0.00) |
| Other(%) | 981(39.65) |
| AE occurrence time—medication date (days) |  |
| 0-30d(%) | 244( 9.86) |
| 31-60d(%) | 90( 3.64) |
| 61-90d(%) | 77( 3.11) |
| 91-120d(%) | 46( 1.86) |
| 121-150d(%) | 29( 1.17) |
| 151-180d(%) | 40( 1.62) |
| 181-360d(%) | 118( 4.77) |
| 360d<(%) | 136( 5.50) |
| Not Specified(%) | 1694(68.47) |
